# Who is the next leader? Understanding women leadership development and succession planning in Saudi Arabian higher educational institutions

**DOI:** 10.3389/fsoc.2024.1442543

**Published:** 2024-10-23

**Authors:** Dina Abdullah Dahlan

**Affiliations:** College of Business Administration, University of Business and Technology, Jeddah, Saudi Arabia

**Keywords:** female leadership, succession planning, education, Vision 2030, Saudi Arabia

## Abstract

Leadership development and succession planning is a valuable strategy for closing the gender gap and providing an equal chance for males and females to be trained and involved in leadership roles. This strategy is an incredibly effective tool in higher education, where there is a significant disparity between male and female academic leaders. Despite its effectiveness, succession planning remained an overlooked component of the higher education system. Further research studies enhance our understanding of the current state of succession planning and the factors that affect the recruitment and selection of future academic leaders. Hence, this study aims to identify the current women’s leadership development and succession planning practices in public and private universities in Saudi Arabia. A qualitative study was conducted in a Saudi Arabian higher education setting. The interest in Saudi Arabia stems from the fact that succession planning is less likely to be practiced since gender discrimination is apparent when assigning leadership positions. Results indicate that top powers typically select leaders since succession planning is absent in Saudi Arabia’s higher education institutions. In this circumstance, networking and relationships play a significant role, as these institutions have no formalized succession planning process. In a highly gender-segregated society like Saudi Arabia, women are deprived of expanding their networks and showcasing capabilities to male decision-makers. The results of this study further identified the underlying components of the women’s leadership and succession planning process in the Saudi Arabian setting. This study contributes to leadership and succession planning knowledge and its implications, which may extend opportunities for practitioners, consultants, and policymakers to include women in higher education succession planning.

## 1 Introduction

Previously, educational institutions focused on academic performance and policy development to achieve competitive advantages (White, 2003; Chance, 2022). Recent trends in education shifted the dynamics to women’s leadership (Maheshwari and Nayak, 2022), which played an essential role in achieving long and short-term goals (Madsen, 2012). The landscape of women’s leadership in higher education has been enduring significant transformation. Despite historical gender discrimination and cultural constraints, there is a growing recognition of the importance of female leadership in driving innovation and academic excellence (Phillips, 2021). Chance (2022) stated that “women are nearly three times more likely to aspire to senior leadership with prestigious titles than their counterparts” (p. 45).

Empirically, studies, for example, Luna (2012) highlighted that the strategic leadership efforts by women ensure they are more productive and competitive, build trust with communities, and foster a collaborative and enabling environment to enhance learning practices. Recently, Moodly (2021) investigated and concluded that women play an important role in educational leadership, mainly bringing innovation within academic institutions and fostering diversity and inclusivity. Most importantly, in this era of modernization and digitalization in the educational system, succession planning is an essential strategic tool that ensures the sustainable development of women’s leadership in academic institutions (Maheshwari and Nayak, 2022). Nonetheless, educational institutions with gender-diverse leadership teams benefit from enhanced strategic decision-making, maximize organizational performance, and confirm the academic outcomes of teachers and students (Chance, 2022). For instance, a study by Alqahtani (2021) found that educational institutions with women presidents and succession planning frameworks often prioritize inclusive policies and practices, leading to a more supportive academic environment for staff and students.

Succession planning facilitates dynamic progress by offering vast targeted leadership development opportunities, career advancement pathways, and mentorship programs (Raby and Valeau, 2021). Research conducted by Phillips (2021) highlights that educational institutions with effectual succession planning are more likely to promote women to senior positions, thereby addressing gender imbalances and fostering a culture of equality. One effective way to expand the internal pool of leadership candidates and fill leadership positions is by developing and implementing succession planning programs (Fusarelli et al., 2018).

Prior studies suggested that the succession plan should be evaluated using its critical metrics, including the number of employees on the succession plan placed in crucial positions over each calendar year, increased employee retention, and enhanced commitment to work and the workplace (Desarno et al., 2021). Previously, succession planning applications in organizations have been widely covered in past studies with a sufficient number of initiatives, strategic designs, and programs for corporate organizations (Rosenthal et al., 2018; Black et al., 2019; Ayandibu and Kaseeram, 2020). Therefore, the concept of the succession plan is still novel in higher education and is hardly addressed by scholarly work in education (Gilbert, 2017; Keller, 2018; Phillips, 2021) and women’s leadership (Alqahtani, 2021), mainly in Saudi Arabia.

According to the Global Gender Index, Saudi Arabia still has a wide gender gap, ranking 141 (out of 149) in terms of creating equal opportunities for men and women (World Economic Forum, 2018). In Saudi Arabia, females are disadvantaged in their practice, and gender disparities occur based on sociocultural and religious factors (Abalkhail, 2017; Fallatah and Syed, 2017). Therefore, because women face challenges holding leadership positions in Saudi Arabia, this study aims to understand female leadership development and succession planning in Saudi Arabian higher educational institutions.

This paper covers several sections. After the introduction, a literature review on succession planning is presented. The following section describes the qualitative methods applied in this study. The themes that emerged from the thematic analysis are then presented. The last section concludes and discusses the implications and limitations which provide direction for future research.

## 2 Literature review

### 2.1 Women leadership

Women are gaining visibility as highly qualified professionals worldwide and are increasingly recognized for their achievements in leading initiatives, organizations, and nations (Bin Bakr and Alfayez, 2022). Alqahtani (2021) defines women’s status as a leader as a global phenomenon not specific to a particular geographical context. To illustrate, Dahlvig and Longman (2020) revealed that women who aspire to senior-level leadership in the United States continue to find their professional journeys hindered by various internal, organizational, and broader cultural barriers. Similarly, Gandhi and Sen (2021) found that female leadership in the top positions in academia in India is not just under-represented but also under-researched. Similarly, in the Vietnam context, Tran (2020) found evidence of the underrepresentation of women, especially in high-level leadership positions. Despite the recent rapid growth in higher education worldwide, gender inequality in higher education remains a significant issue (Olson-Strom and Rao, 2020). Therefore, the findings from key studies on women’s leadership are presented in Table 1.

**Table 1:** Empirical studies on women leadership in education.

Author(s)	Country	Findings	Implications
Madsen (2012)	USA	The findings show the representation of women in leadership roles, the barriers they encounter in career advancement, the impact of gender biases and stereotypes, strategies for promoting gender diversity in leadership, and the implications for organizational effectiveness and equity in higher education settings.	The implications emphasized the importance of advancing gender equality in leadership positions within higher education for the benefit of individuals, organizations, and society.
Maheshwari and Nayak (2022)	Vietnam	Findings offering insights into cultural, social, and institutional contexts significantly shape women’s experiences in leadership roles in higher education.	Study insights into challenges and successes, guiding future policy, research, and cultural shifts toward fostering gender diversity and equity in academic leadership.
White (2003)	Australia	The findings highlight mentorship, supportive organizational cultures, and targeted leadership development programs influence women’s leadership practices.	The article proposes policy recommendations to promote gender diversity and equity in academic leadership, such as affirmative action policies, gender-sensitive recruitment and promotion practices, and family-friendly policies.
Chance (2022)	USA	The study identifies various strategies that Black women in higher education leadership use to navigate and overcome cultural adversity, such as developing strong support networks, engaging in self-care practices, and leveraging their cultural identity as a source of strength.	The study’s implications include recommendations for policy changes at the institutional and governmental levels to address the structural inequalities that Black women face in higher education leadership.
Moodly (2021)	South Africa	The study reveals significant differences in how men and women in leadership positions perceive the challenges and opportunities for women in higher education leadership.	The study advocates for developing and implementing more inclusive policies and practices that promote gender diversity in leadership positions within higher education institutions.

However, educational institutions have begun investing more in leadership programs that encourage both men and women to participate (Kairys, 2018). In addition, building leadership capacity and skills throughout the academic staff, linking resources to a gender-balanced leadership team, and seeding leadership posts in areas of specific gender imbalance are all positive moves to achieve a pool of academic leaders (Power, 2020). Accordingly, White (2003) stated that women leader promotes diversity and inclusion, fostering an environment where different perspectives enhance decision-making and problem-solving. Women leaders are role models, inspiring female students and staff to pursue leadership roles. Such practices of women’s leadership, mainly in cultured divert organizations, help break down gender stereotypes and promote gender equality in traditionally male-dominated fields. Additionally, women leaders often emphasize collaborative and empathetic management styles, which can lead to a more supportive and nurturing educational environment (Maheshwari and Nayak, 2022). This approach benefits students and staff and enhances overall institutional effectiveness and morale.

Practically, the number of women in leadership positions, mainly in developing countries, is still far from equal to that of men (Tran, 2020). Although a few drastic steps have been taken to reduce the gap in gender imbalance in senior positions in higher education, more needs to be done, as the current system is slow and ineffective (Power, 2020). Moreover, a bias toward male leadership in higher education has amplified the gender imbalance (Tran, 2020).

The current research agenda advocates for more female representation and highlights the positive impacts of female leaders on the community. Despite this recognition, women still do not have equal access to opportunities (Tran, 2020). Female leadership is still in its infancy, and more is needed to ensure they are on equal footing as their male counterparts. Unfortunately, females tend to be excluded from organizational policymaking, planning, and decision-making (Mustafa and Troudi, 2019).

In conclusion, women’s educational leadership fosters diversity, equality, and effective management within academic institutions. By promoting inclusive policies and serving as role models, women leaders contribute significantly to advancing and enriching the educational landscape.

### 2.2 Succession planning

Succession planning is one way to identify the leadership capacity possessed by junior staff and a clear career path; this must align with the development planned for future leaders (Yudianto et al., 2023). A succession plan is a deliberate and systematic effort to ensure leadership continuity in key positions, retain and develop future leaders, and encourage professional development and advancement (Jones and Rivers, 2021). In light of such systematic efforts, males and females will be treated equally, having the chance to grow on a career path. Additionally, succession planning is one way to reduce employee turnover because it positively affects employees’ perceptions of possibly taking up higher roles. One of the main factors that results in employees leaving a company is the lack of room for growth (Owolabi and Adeosun, 2021). Accordingly, organizations need educated workers to become successful and retain, grow, and maintain employees’ careers (Malokani et al., 2023).

Therefore, the practice of succession planning is not common in education. When a leader departs, many programs and initiatives often disappear because the structure and motivation that support the programs reside within the administrator instead of being embedded within the staff (Fusarelli et al., 2018). Investment in internal talent is not what most higher education institutions do when looking for a leader (Cavanaugh, 2017), which indicates a lack of succession planning. The main reason for the lack of succession planning in higher education is the reluctance of faculty to take initiative in administrative positions and similar positions (Land, 2003). Moreover, faculties depict a lack of willingness to prepare themselves for higher positions in the academic community due to fear of loss of personal time, handling conflicts, more sacrifice, and extra workload, leading to the creation of less worthy positions (Land, 2003; Keller, 2018). Therefore, the findings from key studies on succession plans in education are presented in Table 2.

**Table 2:** Empirical studies on succession plans in education.

Author(s)	Country	Findings	Implications
Luna (2012)	USA	The study identifies an impending leadership crisis in American higher education due to many senior administrators approaching retirement age.	The article emphasizes the need for higher education institutions to develop and implement comprehensive succession plans to ensure a smooth transition of leadership roles.
Barton (2019)	USA	The study emphasizes the critical importance of succession planning in Christian higher education to ensure organizational continuity and stability during leadership transitions.	The study’s implications could include policy recommendations for governing boards and administrative bodies to institutionalize succession planning processes, making them an integral part of the institution’s strategic planning efforts.
Raby and Valeau (2021)	USA	The study identifies a significant need for position-specific training programs tailored to the unique requirements of community college international education leaders.	The study advocates for implementing robust succession planning models within community colleges to ensure a pipeline of well-prepared leaders ready to take on international education roles.
Phillips (2021)	Georgia	The study found the current state of succession planning in nursing education revealing a lack of structured and formalized processes in many institutions.	The study advocates for implementing structured succession planning programs in nursing education, including clear pathways for career advancement and leadership roles.
Ghazali et al. (2023)	Malaysia	The study focuses on validating a scale designed to measure employee competencies specifically relevant to succession planning for administrators in higher education institutions.	The article emphasizes the need for continuous evaluation and refinement of the scale based on feedback and changing organizational needs, ensuring its ongoing relevance and effectiveness.

Although literature exists from the perspective of succession planning for businesses and corporations, leadership development, and planning for CEO positions, there is limited literature addressing succession planning in higher education. Unfortunately, limited scholarly work has investigated the succession plan in higher education settings, especially in Saudi Arabia. Many researchers, for example, Klein and Salk (2013); Keller (2018); and Ritchie (2020) shed light on the leadership crises and the need for a succession plan in an academic setting; however, these studies need more investigation to fill the gap and provide a structured approach for academic preparedness. Thus, the utmost need is to develop a succession-planning framework for educational institutions to overcome the leadership crisis and create equal opportunities for females and minorities to grow in academic culture. Sustainable succession plans have recently been viewed as an effective tool for providing gender equity in accessing and achieving educational opportunities (Dahlvig and Longman, 2020).

Noticeably, Cavanaugh (2017) suggested two dominant ways to approach succession planning in higher education: (a) succession planning for emergencies and (b) succession planning for predictable leadership transitions. Succession planning for emergencies is essentially a component of disaster or continuity of operational planning. Succession plans for predictable leadership transitions involve creating a pool of internal talents from which a successor leader is chosen through a selection process. In this regard, the present study is not concerned with succession planning for emergencies and only focuses on the second approach to ensure a smooth transition in leadership positions in educational settings.

## 3 Method

### 3.1 Population and sampling

The present study used purposive/judgmental and snowball sampling techniques to collect data from academics and non-academic staff from Saudi Arabia’s higher educational institutions (Saunders and Kitzinger, 2015). The researcher ensured the involvement of the participants in the administration, operation, and academics. These individuals included senior management (i.e., who leads the governance system, plays their role in change management and leadership decisions in universities); rector or senior management personnel (i.e., ensures effective implementation of policies and develops systems when needed); dean (i.e., plays a role in terms of execution of operations); head of departments/schools (i.e., manages regular operations and connects line management with top management); instructors (i.e., delivers and manages academic lecturers and potential candidates for future leadership position in the universities); administrators (i.e., plays a role in managing the record, operationalization of activities, and supports top and middle management in managing tasks as per standard operating procedures); education consultants (i.e., provides education management consultancies to different institutes and helps strategize the operation/business), and senior personnel from the education ministry (that is, ensures the implementation of legislation, governance system, policies in the education sector, and strategizes the national agenda).

#### 3.1.1 Demographic characteristic

Overall, 18 semi-structured interviews lasted an average of 60 min. Demographic information, including gender, position, years of experience, and qualifications, was identified. Since the personal information of the interviewees was confidential, the names were coded from ER1 to ER18, and the name of the university/organization was not mentioned. “ER” stands for Educational Responses, and numbers refer to the order in which interviews were conducted. The respondents’ demographic profiles are presented in Table 3.

**Table 3:** Profile of the participants.

Demographic items		Frequency	Percentage
Gender	Male	8	44.5
	Female	10	55.5
Job position	President	2	11
	Vice president	1	5.5
	Vice-Rector	2	11
	Dean	5	27.7
	Senior Consultant	1	5.5
	Lecturer	5	27.7
	HR Manager	2	11
Job experience	Less than 3 years	1	5.5
	3–6 years	6	33.3
	7–10 years	2	11
	10–13 years	2	11
	Above 13 years	7	38.8
Qualification	Masters	3	16.6
	PhD	14	77.7
	Other/Diploma/Certification	1	5.5

The demographic data provides valuable insights into the composition and qualifications of individuals in leadership positions within Saudi Arabian universities. Table 3 indicates a slight majority of female leaders, highlighting a positive trend towards gender diversity in university leadership roles. Deans and lecturers comprise the most significant segments of the leadership positions at 27.7% each. Next, most individuals have over 13 years of experience (38.8%), with another considerable portion having 3–6 years of experience (33.3%). Finally, the overwhelming majority hold PhDs (77.7%), reflecting a high academic qualification among the leaders.

### 3.2 Data collection

Data collection began, with the participants being contacted via face-to-face meetings or email to explain the study’s purpose, required data, and interview duration. It also confirmed the availability of participants for a specific day and time. This step was supported by encouraging participation in the study and obtaining consent. After receiving initial confirmation from the participants, the researcher emailed relevant protocol documents and requested them to review them in detail and confirm the interview appointment time at their convenience. Later, the researcher followed the confirmed plan via email and was flexible if the plan changed or needed to reschedule the interview.

During the interviews, the researcher recorded all interviews using Google Meet and a voice recorder. The researcher also allowed participants to skip any questions if they felt uncomfortable answering them. Participants were allowed to interrupt the interviewer when clarity was required. The interviewer made this discussion informal, so the participants felt comfortable saying what they thought. The interviewer informed the participants that this discussion had no right or wrong answers. However, participants were right to say anything and document their experiences and thoughts regarding this study. The participant was informed about the interview duration, around 60–90 min. Moreover, the participants’ information was kept confidential and saved on a password-protected computer. Any information about the participant had a number on it instead of their name.

### 3.3 Pilot study

A pilot study was conducted to check the reliability and validity of the questions for the in-depth interviews. The pilot study was conducted at a private university in Jeddah, Saudi Arabia, using the same protocols and sample sizes as in the actual research. The selection of a private university for a pilot study was based on convenience, accessibility, and geographical proximity (Yin, 2003). The study participants included one senior management personnel, one head of department/school, and one instructor. All interviews were conducted privately to avoid participation distraction and maintain confidentiality. During the pilot, it was found that it was necessary to introduce the research aim. Starting interviews without an initial explanation confused the participants and hardened the interview process.

Additionally, it was found that language barriers must be taken into consideration. One participant was willing to be interviewed in English but had difficulty expressing their ideas fluently. In such cases, the captured information was neither rich nor reliable because understanding some points was complex and challenging. To avoid such problems in the following data collection phase, the researcher conducted interviews in Arabic with those with weak English communication skills.

### 3.4 Data analysis

This study used a qualitative thematic approach to analyze the data. Qualitative designs provide valuable insights for documenting a selected population’s values, behaviors, and opinions. The data analysis procedure is based on the verbatim report and manual transcription after the interview with study participants. In addition, the researcher used a thematic content analysis approach to develop themes aligned with the research objectives. This step helped the researcher reduce errors, mistyped, misinterpreted, and missing words, causing extra efforts to manage data sets. The final transcripts were transferred into NVivo 12 plus to summarize data and derive conclusions. In this regard, the replication favored the researcher because it supports the study findings and reduces the interview data’s biases and interpretations. The coding structure was used manually and with the support of NVivo steps. Thus, preliminary codes (i.e., themes, categories, concepts, properties, codes) were identified via constant comparative analysis. This approach helped the researcher to get initial support to finalize the codes and add new ones if needed. In addition, unwanted information was pulled out and replaced with dots “…” to truncate excessively descriptive data. In a qualitative study, Creswell and Clark (2007) identified three coding phases: open, axial, and selective coding. Open coding establishes an initial connection between these categories. Axial coding supports dimensions from the categories and results to produce a contextual story. Selective coding helps in theoretical formation. This study used open coding while reading and highlighting the transcripts in NVivo. In the second phase, the researcher synthesized the codes obtained in the previous phase and placed the open code under a broader category. In the last phase, the researcher attempted to understand the relationship between the sub-themes, integrate similar ones, and list them under a core category. For instance, the skill inventory identified in axial coding can be categorized under critical components of succession planning. After obtaining the themes and sub-themes, the final report was written and is presented in the following section.

## 4 Findings

This study aims to explore and understand female leadership development and succession planning in Saudi Arabian higher educational institutions. The emerging themes cover (a) the definition of succession planning, (b) the presence of succession planning in universities, (c) the selection of future leaders, and (d) the underlying components of succession planning.

### 4.1 Theme 1: definition of succession planning

The findings from this theme demonstrate that succession planning remains an insufficiently comprehended notion in Saudi Arabia’s higher education institutions. Out of 18 participants, merely four were able to describe this concept. Specifically, ER5 defined succession planning as a strategy that ensures the replacement of a suitable employee when someone leaves a key position.

*“Succession planning is a strategy for passing on leadership roles, often including company ownership, to an employee or a group of employees. This ensures businesses run smoothly after their most important people move on to new opportunities, retire, or pass away. This process is also known as replacement planning (ER5).”*

Another participant associated succession planning with the HR department, ER1, affirmed that:

*“Succession planning is an HR system that defines the process of replacing key position holders upward or downward throughout the organizational ladder (ER1).”*

The following participant defined succession planning as ensuring business continuity by identifying and preparing employees suitable for future managerial positions. Succession planning is selecting, developing, and preparing high-performing employees with the right skills and knowledge to take over the management’s position at the right time. This includes different types of job training, coaching, and mentoring. Performance evaluation is a crucial part of the succession-planning process.

*“Succession planning is crucial to ensure business continuity and leadership development. It also motivates employees to enhance their organizational citizenship (ER6).”*

One of the current HR managers refers to succession planning as activities preventing organizational disruption when a critical position becomes vacant. Additionally, the benefits and importance of succession planning are explained: from one side, organizations would ensure workflow continuity, and on the other hand, employees will be more motivated and satisfied due to a clear career path. Succession planning is one of the essential activities that organizations must focus on to ensure the continuity of their work in the required manner and to avoid any disruption to the workflow in case of a vacancy in critical positions in the organization.

*“It is important to identify critical jobs and candidates for these jobs to develop and train them to become suitable for this position without any disruption in the workflow or waste of money or time (ER18).”*

Notably, demographic factors like age and education significantly influence women’s leadership opportunities within quota systems. One respondent noted that in Saudi Arabia, young women who have recently graduated from college encounter more challenges securing leadership positions than their more experienced counterparts.

*"Younger women with limited or no experience, mainly undergraduate, often face greater challenges in attaining leadership positions within organizations (ER5)."*

Furthermore, a respondent indicated that external factors such as religious expectations, cultural norms, and government policies on local and expat significantly influence women’s leadership practices in private and public organizations.

*"Local and foreign women face significant challenges in attaining leadership positions, particularly within family businesses. Religious factors also pose substantial barriers to women's leadership participation (ER4).”*

Furthermore, the respondent added that the government is initiating policies to empower women and provide them with maximum opportunities.

*“Recent government policies centered on women’s empowerment have created new opportunities, shaping a dynamic landscape for women leaders in Saudi Arabia. While progress is evident, significant challenges persist (ER4).”*

Based on the above-stated descriptions, succession planning in Saudi Arabian higher education can be defined as succession planning, which involves selecting and preparing suitable employees to hold leadership positions at the appropriate time. This strategic plan, developed by the HR department, ensures business continuity when a leader moves on to new opportunities, retires, or passes away. Training and coaching are inseparable parts of the plan while preparing future leaders.

### 4.2 Theme 2: presence of succession planning

Not surprisingly, the analysis of interviews with academics revealed that female succession planning is absent in Saudi Arabia. It can be confidently stated that there is no formal succession plan for identifying, preparing, and appointing female leaders within the country. The following participants highlighted that:

*“Unfortunately, we do not have succession planning … We do support our employees in the HR department, but we do not have a specific process for this … We never know when an academic employee is about to be hired; we might know that someone is already hired (ER18).”*

*“You can see that, all the time, the females are trying to prove themselves, but there is still no career development plan in the HR department. There is no formal succession planning … they randomly select the right person (ER6).”*

The following statement concerns the lived experiences of ER3, who has 28 years of working experience and currently holds a senior consultant position in the Ministry of Higher Education. He never observed any programs that were mainly designed for female leadership.

*“Frankly, there are no, or I did not notice, programs specially designed to prepare females to be leaders. There are no studies specifically designed for women’s leadership at universities. I have worked for many years at the university and the Ministry of Education … There is a shortage of such programs in universities and the Ministry of Education (ER3).”*

A former university president shared that, although he supported female leaders and brought a systematic selection process to ensure equal career growth, he did not document any of them.

*“I did not get the chance to formulate this into a plan for succession … it is still a young movement not yet to be regulated to put into forms or plans, and it was just running, so I did not get into that level or stage of assuring the sustainability of this initiative (ER16).”*

This statement is also highlighted by Fusarelli et al. (2018), specifying that many programs and initiatives disappear when a leader departs because the structure and motivation that support the programs reside within the administrator instead of being embedded within the staff.

### 4.3 Theme 3: selection of future leaders

In the absence of succession planning, the credibility of the process for identifying and selecting future leaders is questionable. This study delves deeper into how educational leaders are chosen in Saudi Arabia. The thematic analysis of the transcripts demonstrated that top-level executives mainly rely on relationships, networks, and trust when making these selections. That is, the decisions of the top powers, including the president and board of trustees, determine who will hold a leadership position.

*“Of course, the idea of top management is the deciding factor in who will hold that position. (ER1).”*

“*The university president chooses those who want them to lead the university. It is based only on the president to select the vice president (ER8).”*

*“The president is elected by the Minister of Higher Education, not us. The president chose the vice Chancellor and members. Senior management chooses and elects deans. (ER15).”*

While explaining the selection of future leaders, some interviewees also referred to the criteria by which the supreme powers of the organization choose future leaders. For instance, the following participant highlighted that networks and relationships matter when selecting leaders.

*“Some candidates are qualified and have high capabilities, but they do not have social relationships that would help them get nominated or recommended to be leaders (ER3).*

*“… here all the positions, most of the positions are held by males, not even in Saudi Arabia but the whole world is based on the relationship (ER11).”*

*“I got employed by somebody who knew me, and I did not go through the HR; it was an appointment; I did not apply for a job; I got appointed (ER4).”*

In addition to networks and relationships, trust was a criterion for selecting leaders.

*“… if the vice president knows somebody, he trusts them, so trust is before anything, before skill, before anything … they always say skill comes later, I can train them (ER10).”*

### 4.4 Theme 4: underlying components of succession planning

Establishing effective succession planning can guide organizations to evaluate their present situation and decide the critical employees who will ultimately step into leadership and senior management positions (Desarno et al., 2021). However, before practicing succession planning, it is imperative to identify and comprehend the main components that affect the development of succession planning to ensure that women are fully considered in the selection process. The investigation of the interviews revealed that these factors are classifiable into training programs, proactive human resource departments, designing job descriptions and career paths, skill inventory, quota policies, and organizational support.

#### 4.4.1 Sub-theme 1: training program

The interviewees identified training as the most frequently cited element of the abovementioned sub-themes. Many participants acknowledged that training is an inseparable part of succession planning. This theme refers to preparing a pool of people who have potential and are willing to take a leadership position when there is a vacancy. The preparation can be conducted through courses, workshops, conferences, mentoring, and coaching sessions. According to Desarno et al. (2021), mentoring and coaching, internal and external educational opportunities, position observation, assessment, and feedback are essential parts of the process. To exemplify this notion further, the following quotes indicate that when employees are well-trained, they will be ready to hold different leadership positions based on the competence level required for filling vacant positions.

*“The training targets a group of people rather than one specific person … you need to have the luxury of choosing people, looking at their qualifications, and then deciding who will fit the position. (ER14).”*

*“… providing training to develop skills. I think the person has to have some kind of guidance or a mentor to help them through these strategies (ER11).”*

It is mentioned that the provision of training encourages females to hold leadership positions. Additionally, training and educating employees is a way to face resistance and helps individuals accept the development of succession planning.

*“As for encouraging females to hold such positions, providing training courses could help (ER3).”*

*“We have to educate people, and we have to hold seminars and meetings … so when we explain that they will support it, there will be no more resistance (ER9).”*

#### 4.4.2 Sub-theme 2: proactive human resource department

The second theme was having a proactive human resource department designing, formulating, and implementing succession planning initiatives. Several participants claimed that successful implementation of succession planning in universities relies on an active HR department that collaborates with other units and continuously interacts with employees, as quoted below.

*“Developing a mature HR department with the right capabilities to design succession planning… HR should work with immediate supervisors and managers to help identify future leaders (ER6).”*

“*In this regard, the HR department should be a contributor (ER12).”*

*“They have to come up with a comprehensive development plan … maybe they can hire consultants for HR to help them with their succession plan. (ER13).”*

#### 4.4.3 Sub-theme 3: designing job description and career path

In succession planning development, it is essential to ensure job descriptions and career paths are well documented. While a clear job description elucidates the responsibilities of the employees, a structured career path motivates employees by letting them know about the positions they will hold as they grow in the field. The following participant expressed discontentment regarding the ambiguous clear career path and lack of opportunities for promotion:

*“A clear job description should be available. Job description, employees with different majors, and performance evaluation are all important elements. We do not have a clear career path (ER18).”*

A clear career path is crucial for successful succession planning, as it helps employees know their responsibilities and estimate where they will be within the next few years. ER1 explains this well:

*“We can specify within the job description we tell the individual that these are your responsibilities; these are your relationships with others, and this is your end of the line. Where will you be five years from now, nine years from now, and nineteen years from now? If we did that, we could have a successful succession planning (ER1).”*

#### 4.4.4 Sub-theme 4: skill inventory

Skill inventory is another crucial component that refers to a specific platform encompassing a list of employees’ capabilities and the skills required for each job position. Utilizing this kill inventory helps set the standards for selecting and prioritizing suitable candidates through a designed system. One human resource manager recalled his experience with skill inventory generation, documenting the current employees’ skills while listing the necessary skills for each leadership position. Subsequently, the human resource department prepares employees whose skills align with specific leadership positions.

*“We built a specific skill inventory for each position … what the skills are needed for each employee at each department then to nominate and develop specific leaders to handle those departments later on (ER17).”*

Another participant elaborated that setting the criteria was an essential element. First, the skills and qualifications required for a particular position should be clarified; then, the best candidate who fits the position will be chosen from the pool of candidates.

*“We should have criteria, we give it weight, and then we select candidates and prioritize them through a blind system instead of who knows who. We need to develop criteria to select the candidates (ER2).”*

#### 4.4.5 Sub-theme 5: quota policy

Thematic analysis indicated that quotas could be a key element in facilitating succession planning. It appears that a quota is a tactic to provide more opportunities for females to be involved in leadership roles. Setting a quota creates avenues for more female representation in leadership positions and closes the gender gap. As a result, there will be a pool of qualified female candidates, and succession planning to include females will be less challenging.

*“… the only way right now is to impose a quota on the university council. I didn’t see another way it’s imperative to put quota … I will not sign any order to form a committee in the university that at least %30 of these members are females (ER16).”*

As Dahlvig and Longman (2020) emphasized, expanding women’s representation in leadership is necessary across all sectors, and quota policies are a pathway to achieve numerical parity. Hence, quotas should be considered to address gender inequities in higher education boards and senior leadership teams. The previous quote states that at least 30% of university councils must be female.

*“… the university council must have one-third of women (ER2).”*

Similarly, one participant expressed a positive attitude toward implementing quotas. He emphasized that a quota is a way to implement succession planning in societies with gender discrimination successfully.

*“… to reduce the gender bias, they put a quota in terms of positions within an organization … If you have that quota and everybody can accept that the succession planning for females would be even easier (ER7).”*

#### 4.4.6 Sub-theme 6: organizational support

None of these elements can be practiced without complete backing from the organization or its highest-level executives. It is, therefore, imperative to ensure that top management is aware of the advantages and benefits offered by this plan and provides financial and nonfinancial assistance. As pointed out by ER9, top management must understand the notion in-depth.

*“… upper management must understand that and support it … if it’s supported by upper management, everything is going smooth (ER9).”*

One HR manager who spent a few years developing initiatives and a systematic replacement of future leaders referred to the critical role of top management in supporting and empowering HR to succeed in decisions and activities.

*“When it came to the HR, it was so simple when I left it a system with many functions, and that could not happen without the support and empowerment from top management (ER17).”*

## 5 Discussion

Succession planning is a strategic process that identifies and develops future leaders to ensure seamless leadership transitions and organizational continuity. In the context of Saudi Arabian universities, succession planning is essential for fostering female leadership and addressing gender disparities in higher education. Structured succession planning within these institutions is pivotal for nurturing female leaders. This approach involves systematic identification of leadership potential, providing training and development opportunities, and ensuring a clear pathway for career advancement.

Selecting future leaders involves a rigorous and transparent process to identify individuals with the potential to ascend to higher leadership roles. This selection is based on merit, performance, and the alignment of individual skills with organizational needs. Ensuring gender diversity in this selection process is critical to promoting female leadership. Effective succession planning comprises several vital components. Comprehensive training programs equip potential leaders with the necessary skills and knowledge. These programs should be tailored to address specific competencies required for university leadership roles.

A proactive HR department is crucial in implementing succession planning by identifying potential leaders early, providing continuous support, and facilitating professional development opportunities. Clear job descriptions and well-defined career paths help aspiring leaders understand the expectations and progression within the organization, ensuring they are prepared for future leadership roles. A skill inventory helps assess current capabilities and identify gaps that need to be addressed through training and development.

In addition, the findings revealed that there would be different levels of acceptance and refusal in various universities depending on organizational culture and geographical location. For instance, a university in Jeddah might perceive female leadership differently from one in more restricted or conservative areas such as Bisha. During the interviews, some participants claimed that their university has no issue accepting female leaders, but others highlighted that the university culture is not ready to receive them. Interestingly, it is also found that acceptance highly depends on a leader’s competency and capability. The more qualified leaders will gain the respect and approval of the employees by proving their capabilities and showing extraordinary performance. In addition, the findings demonstrated that acceptance of female educational leaders depends on factors, i.e., background and personality, organizational culture, leader’s capability, and geographical location. The mentality, personality, age, and educational background of the people play a huge role in accepting the involvement of females. For instance, a senior-aged man raised in a conservative culture and family probably will not agree to be led by a female, primarily because he used to see males as leaders. However, acceptance would be easier for senior-aged people who were educated abroad.

A quota policy can also ensure a balanced representation of women in leadership positions, promoting gender equity. Organizational support is vital in creating an environment where female leaders can thrive, including mentorship programs, networking opportunities, and institutional commitment to gender diversity. Overall, these components work together to create a robust succession planning framework that empowers female leaders and supports the strategic goals of Saudi Arabian universities.

### 5.1 Theoretical and practical implications

Women face significant barriers to reaching top management and leadership positions across nearly all professions, including higher education (Semela et al., 2020). This ongoing gender disparity in leadership roles underscores the urgent need for effective measures to promote gender equality and social justice in higher education leadership (Tran, 2020). The persistent leadership gaps have highlighted the importance of succession planning (Keller, 2018), facilitating a smooth transition into leadership roles. While succession planning is a common and expected practice in the business world, it has often been overlooked in the context of higher education (Phillips, 2021). Thus, this study is crucial in advancing the academic discussion on succession planning, addressing existing gaps, and establishing meaningful connections between female leadership and this essential organizational process.

Beyond its theoretical contributions, this study holds significant practical implications. The findings can guide decision-makers in shaping educational policies, highlighting necessary changes, and modernizing educational practices and leadership methods to achieve desired educational outcomes (Al-Jaradat, 2014). Furthermore, the succession plan for the Saudi Arabian context should incorporate a continuous and effective training system, along with personal mentoring, to cultivate leadership skills and provide unique growth opportunities for Saudi women.

The findings of this study emphasize the importance of universities establishing a solid and clear career path and job descriptions, which can significantly enhance employee motivation. Additionally, it is crucial to allocate financial resources for incentives and compensation. Monetary benefits can motivate women, who often juggle work and family responsibilities, to engage more actively in succession planning. This approach can reduce reluctance and increase the willingness of potential female leaders. It is also essential to consider that manually generating and updating employee skill inventory is impractical. Instead, universities should implement a system to store and update information as positions and employees change. This database should include the skills of current employees and the skills required for each leadership position. Once the skill inventory is established, suitable training courses and mentoring sessions should be offered to a pool of candidates to prepare them for future leadership roles.

Overall, before selecting the leaders and avoiding bias and gender discrimination, the universities may allocate a quota to include females in leadership roles. As indicated before, in a highly segregated higher educational setting, males will have a higher chance of being nominated for leadership positions if a quota is not considered for females. Lastly, the entire process must be evaluated based on performance and feedback, and necessary amendments must be made.

## 6 Conclusion, limitations, and recommendations

Succession planning is a critical process that universities must undertake to promote inclusivity, diversity, and equal opportunities for men and women. Succession planning allows for effective leadership transitions by identifying and developing key talent and providing them with growth opportunities. This qualitative study aimed to explore how this practice is being implemented in Saudi Arabian higher education, where gender discrimination remains challenging. Before examining the current state of succession planning, the research sought to understand how academicians are perceived and define this concept in Saudi Arabia. Although many participants were unfamiliar with this notion, few others pointed out that succession planning is selecting and preparing future leaders by training employees and helping them learn and improve their leadership skills. It is also recommended that the HR department should be responsible for formulating and executing succession planning. As expected, the findings of this qualitative study revealed that succession planning is absent in Saudi Arabian higher education institutions. Instead of selecting leaders based on a systematic plan, they are chosen mainly by top powers based on networking and personal relationships. This approach limits opportunities for females to demonstrate their capabilities to predominantly male decision-makers. Key factors affecting the succession planning process have also been highlighted in Saudi Arabia’s context. The thematic analysis discovered that training programs, proactive human resource department, job description and career path, skill inventory management, quota policy, and organizational support are the key components of succession planning that should be considered when establishing succession planning in Saudi Arabia.

During our current research, several constraints were identified that could potentially provide insights and guidelines for forthcoming studies. The initial limitation of this study pertains to the fact that this investigation is solely carried out in Saudi Arabia’s higher education system, raising concerns about its generalizability and applicability to other sectors. Second, this qualitative study’s relatively limited sample size restricts the application of findings to other contexts and makes generalizing these findings even within Saudi Arabia doubtful. To address these limitations, conducting analogous investigations in different contexts and considering a larger sample size by encompassing a broader range of higher education institutions within Saudi Arabia would be beneficial. Additionally, employing mixed methodology would be helpful to overcome shortcomings stemming from adopting a qualitative approach. Lastly, this current study is limited by the scope of the investigation. We were mainly concerned with understanding the current state of succession planning, how future leaders are chosen without succession planning, and identifying this plan’s main components. Therefore, future studies may attempt to expand and find challenges to implementing this plan and tactics to conquer challenges and understand how an effective succession plan can be developed and implemented. It would also be valuable to examine the influence of succession planning on several factors, such as turnover intention (Ali and Mehreen, 2019), individual performance, gender segregation, and career development. Another area for further research could be to examine the impact of gender-segregated work environments in different cultural settings on female leadership opportunities (Alomair, 2015).

## Data Availability

The raw data supporting the conclusions of this article will be made available by the authors, without undue reservation.
